# Why Do I Get Side Effects? Personalized (N-of-1) Trials for Statin Intolerance and the Nocebo Effect

**DOI:** 10.1162/99608f92.abc57f1b

**Published:** 2022-09-08

**Authors:** James Philip Howard, Frances A. Wood, Darrel P. Francis

**Affiliations:** 1 National Heart and Lung Institute, Imperial College London, England, United Kingdom

**Keywords:** statins, N-of-1 trials, side effects, nocebo, drug intolerance

## Abstract

The ability of statins to reduce the morbidity and mortality of cardiovascular disease has ensured that they are among the most prescribed drugs in modern medicine. Unfortunately, most patients who start taking statins will end up stopping them, most commonly due to side effects. Confusingly, however, in blinded placebo-controlled trials, side effects appear no more common in those taking statins than those taking placebo. One possible explanation is that ever-present background symptoms are being falsely attributed to statins. However, another explanation is the nocebo effect, where the act of just taking a tablet (even a placebo) causes genuine side effects in patients.

Two recent randomized placebo-controlled personalized (N-of-1) trials have been reported: StatinWise and SAMSON. In these trials, each participant was randomized to multiple periods of statin and placebo, with regular symptom burden assessments. Together, these trials support the existence of a significant nocebo effect from taking statins. Possibly even more importantly, they demonstrate the ability of personalized trials to inform and empower patients: up to half of the patients in these trials were able to successfully restart statins after taking part, despite previously having been statin intolerant. StatinWise and SAMSON have raised public awareness of the nocebo effect in statin intolerance. However, they also demonstrate a potential role for the personalized design outside of clinical trials. If taking part in these personalized experiments allows half of our patients to successfully restart life-saving medications, maybe we should be able to prescribe personalized experiments to our patients in the clinical setting?

## 1. Statins—Their Risks and Benefits

Statins are medications that work to inhibit the enzyme HMG-CoA reductase and thereby greatly reduce the synthesis of cholesterol by the liver. This results in the liver upregulating its expression of the low-density lipoprotein (LDL) receptor, which increases its rate of removal of LDL (a form of ‘bad’ cholesterol) from the blood (Mach et al., 2020).

The effectiveness of statins is dramatic, with reductions of >50% of blood LDL cholesterol a common target for statin therapy. Meta-analysis including over 170,000 patients has demonstrated unequivocal benefits from statins (Cholesterol Treatment Trialists’ (CTT) Collaborators et al., 2012), with reductions in major vascular events of over 20%.

However, despite these benefits, many patients and doctors have concerns about statin therapy. Indeed, the majority of patients starting statins end up abandoning them, most commonly due to side effects (Stroes et al., 2015)(Toth et al., 2019). There are certainly some rare, severe conditions, such as rhabdomyolysis, a breakdown of skeletal muscle cells, which occur more commonly in people taking statins. An analysis of 30 randomized controlled trials of 83,858 patients showed that five people not taking statins experience rhabdomyolysis, and this number increased to seven among those who were taking statins (Thompson et al., 2003).

Far more commonly, doctors encounter the phenomenon of statin tablet–associated muscle symptoms (SAMS). Unblinded observational studies have reported muscular pain and tenderness (myalgia) affecting up to 10–15% of patients (Bruckert et al., 2005)(n.d.).

These ‘real-world’ studies of statin side effects make sobering reading. However, the remarkably common rates of side effects they report are difficult to reconcile with the data from blinded randomized controlled trials (RCTs). A meta-analysis of blinded statin RCTs including more than 80,000 patients showed that patients more commonly had to stop taking *placebo* tablets than statins, due to side effects (Finegold et al., 2014).

It seems difficult to explain this disparity. One possibility is that myalgia is very common regardless of statin therapy, but when it occurs in people on statins, it is incorrectly attributed to the statin. However, another possible explanation is the nocebo effect. In the same way that the placebo effect can mean an inactive tablet is effective at treating adverse symptoms if the patient believes it will help them, in the *nocebo* effect, taking an inactive tablet can make a patient feel much worse, if they expect it to. Because patients in RCTs don’t know if they are receiving statin or placebo, both groups would suffer similar amounts of myalgia from the nocebo effect.

## 2. The Ecological Fallacy and Personalized Trials as a Solution

When a patient comes to a clinic complaining of severe muscle symptoms on statin, the blinded-RCT data provide little reassurance to the doctor and provide little reassurance to the doctor and possibly frustration for the patient. The data do not explain their personal suffering, and could even be considered insulting, if it is interpreted as an accusation that they are lying.

It is for this reason that there has been interest in N-of-1 trials. By providing the patient multiple periods of taking statins and taking placebo, and continually assessing their symptoms, we should be able to accurately gauge a patient’s symptom burden, and how much worse it is taking a placebo than just a statin.

Two recent N-of-1 trials have been undertaken in the United Kingdom with this idea in mind and have been publicized widely: StatinWISE (Herrett et al., 2021) and SAMSON (Howard et al., 2021).

## 3. StatinWISE and SAMSON Trial Designs

The StatinWISE and SAMSON trials are superficially similar. They are both personalized trials that recruited patients with previous statin side effects, and they aimed to identify whether these symptoms were (1) reproducible and (2) worse than any symptoms reproduced by a placebo. They share a common ‘N-of-1’ design, which aims to use precision medicine to enhance outcomes at the patient level (Davidson et al., 2018, December 10)(Vohra, 2016).

Among the differences in their design, listed in Table 1, arguably the most important is that SAMSON included ‘no treatment’ periods. The purpose of this was to allow measurement of not only the increment of symptoms between placebo and statin (i.e., the symptoms truly caused by the statin contents of the tablet) but also the increment of symptoms between taking nothing and taking a placebo (i.e., the nocebo effect).

### 3.1. Findings

In both trials, it was remarkable how small the differences were between symptoms on statin and placebo. Indeed, even though both trials focused on patients who felt they were getting powerful statin side effects, neither trial found a significant difference between statin and placebo.

**Figure 1.**
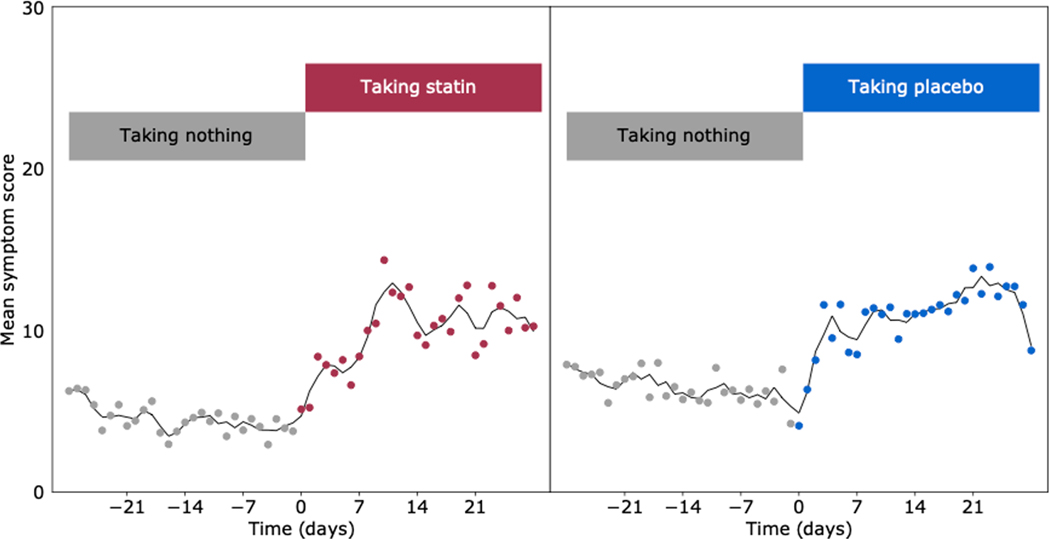
The change in symptoms averaged over all patients when they transition from either no tablet to taking a statin (left) or no tablet to taking a placebo (right) within the SAMSON trial.

From the data science perspective, an interesting feature was the striking similarity between the patterns of symptom onset and regression between the two types of tablets. Figure 1 shows the onset kinetics of symptoms in the SAMSON trial, averaged across every transition between no tablet and tablet, and across all patients, with statin in red and placebo in blue.

**Figure 2.**
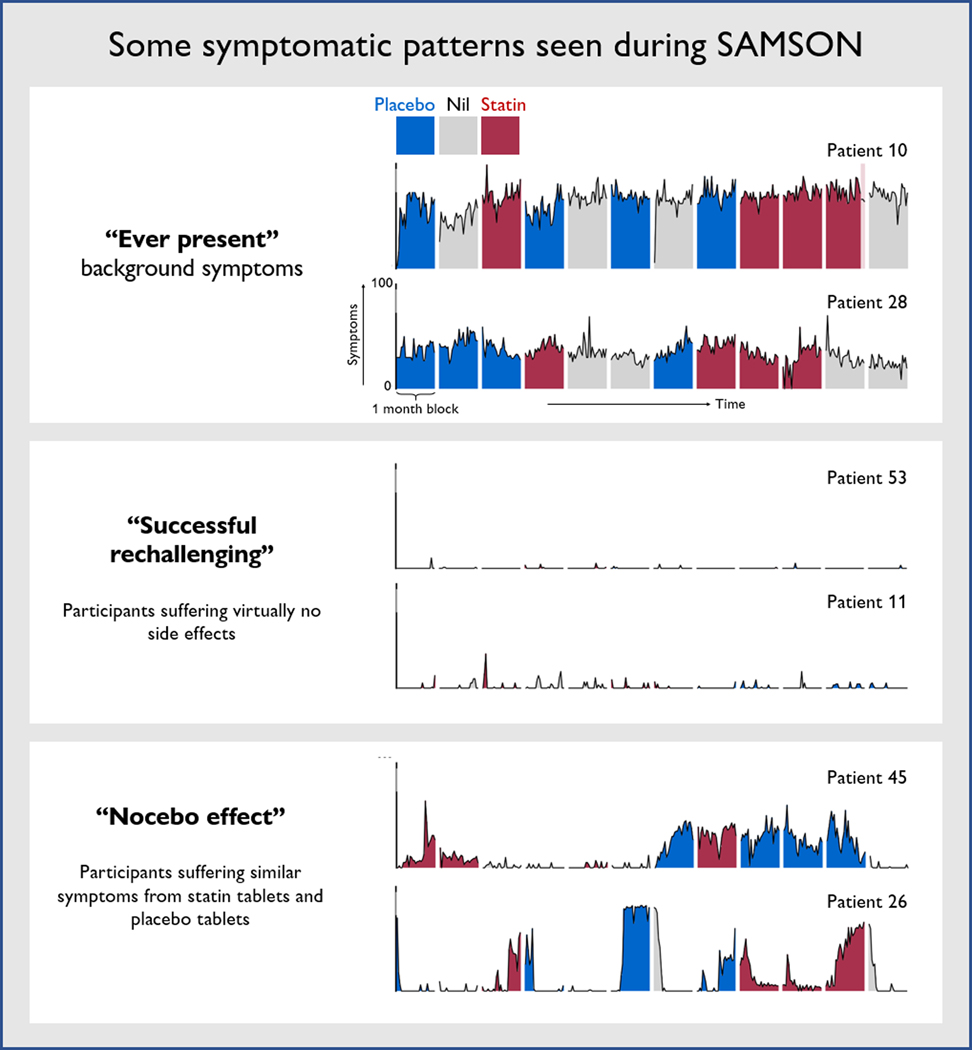
The complete scores from six participants who took part in the SAMSON trial. The horizontal axis represents time, with each of the 12 colored blocks horizontally representing a month of treatment (red for statin, blue for placebo, and grey for no treatment). The vertical axis represents the severity of symptoms. The light red shaded region in Patient 10’s penultimate month indicates that their symptoms were so severe they chose to stop taking tablets early for that month—this was accounted for in the trial analysis.

The personalized design also allows phenotyping of each patient’s symptom patterns. Figure 2 shows the full annual data of six patients from SAMSON who have different patterns. Each patient provided 12 months of data: 4 statin (red), 4 placebo (blue), and 4 no treatment (grey).

The top two patients (10 and 28) have chronic symptoms that are not greatly affected by the tablets. Both patients abandoned statins in the past, because they had assumed that the statin was the driving force and was making them intolerably ill within a week or so of starting. The middle two patients (53 and 11) discovered through participation in the trial that the symptoms that they had previously experienced on statins were not in fact reproduced in the trial. The final two patients (45 and 26) were arguably the most interesting, showing reproducible worsening of symptoms on tablets. However, this worsening was similar between statin and placebo. We infer from this that it was a nocebo effect. The lack of daily symptom scoring in StatinWISE unfortunately means we are not able to see if these three different symptom patterns were seen in that trial, however.

### 3.2. Medical Implications

The results of StatinWISE and SAMSON help doctors and patients understand the paradox of almost no side effects beyond placebo in the blinded RCTs, and yet a frequently intolerable burden of side effects in unblinded clinical practice. They show that the side effect burden in unblinded clinical practice can arise from any of four sources:

#### 3.2.1. Source 1: ‘Ever Present’ Chronic Background Symptoms

When patients are asked about symptoms on statins, they correctly describe them. Because of the prejudice that statins cause such symptoms, and the absence of formal documentation of symptom intensity with and without statins, the symptoms are, understandably, attributed to the statins. This is seen in patients 10 and 28 in SAMSON (Figure 2).

#### 3.2.2. Source 2: Intercurrent Conditions Inducing Symptoms

Even though these occur stochastically, patients and clinicians often conduct informal experiments, with disastrous consequences. When the symptoms are most intense, the statin is stopped, and the symptoms can *only* improve, termed regression to the mean (Morton & Torgerson, 2003). Conversely, the statin is only restarted when the symptoms have resolved, and therefore can only get worse. They therefore find a convincing association between starting and stopping statins and the onset and offset of symptoms. However, this association is purely an artifact of these experiments not having a preset schedule.

#### 3.2.3. Source 3: Nocebo Effect

For reasons that are not clear, some people have developed a physical reaction from taking a tablet that they believe could be a statin. It is also not an imaginary symptom. It is just as real as the palpitations and sweating one would feel if threatened by an armed robber—there is a genuine biological response. This is seen in patients 45 and 26 in SAMSON (Figure 2).

#### 3.2.4. Source 4: True Pharmacological Side Effect

This would become evident from the symptoms on statin being higher than those on placebo. SAMSON and StatinWISE suggest that this is a small proportion, even among patients who had to give up statins because of intolerable side effect. Patient 43 in the Supplementary Appendix of the SAMSON manuscript (Howard et al., 2021) may be an example of this.

## 4. Personalized Trials Are the Beginning of the Story, Not the End

Almost all conventional clinical trials are done to calculate an average effect across all patients, with the intention of applying the findings globally to all similar patients. However, we would argue that N-of-1 designs should be thought of more as a new form of medical practice, where detailed individualized data sets are constructed for each patient, to enable the patient and physician to come to a personalized conclusion. SAMSON showed that by taking part in such a trial, 50% of patients with previously intolerable statin side effects were able to restart these lifesaving drugs. This new task of marshaling and displaying detailed data on an individual, for the perusal of that individual, is an opportunity for data scientists to contribute to saving lives. It was the ability of these trials to show patients their personal side-effect profile that allowed half of them to get back on these lifesaving drugs and still be taking them 6 months later.

## Figures and Tables

**Table 1.** Key differences between the two personalized trials investigating statin side effects.

	StatinWISE	SAMSON

Patients randomized	200	60
Must have stopped statins?	No—could be on statins but considering stopping.	Must have stopped statins due to side effects.
Symptoms	Muscle symptoms	Any symptom leading to statin discontinuation
Treatment blocks	3 statin periods 3 placebo periods	4 statin periods 4 placebo periods 4 ‘no treatment’ periods
Treatment block duration	2 months	1 month
Symptom scoring method	Visual analogue scale (0–10)	Visual analogue scale (0–100)
Symptom period analyzed	Last 7 days of month	Entire month
Primary outcome	Difference in symptom scores	Nocebo ratio*
Assessment of impact on subsequent statin taking	Patients were asked at the end of the trial whether they were intending to restart statins.	Patients were asked 6 months after completing the trial whether they were now taking statins.

* The SAMSON trial planned to report its results in terms of a ‘nocebo ratio,’ but statistical oversights invalidated this approach, and the study authors also presented the difference in symptom scores alongside this.
